# Personal Values in Relation to Risk Intelligence: Evidence from a Multi-Mediation Model

**DOI:** 10.3390/bs11080109

**Published:** 2021-07-31

**Authors:** Andrea Zammitti, Angela Russo, Giuseppe Santisi, Paola Magnano

**Affiliations:** 1 Department of Educational Sciences, University of Catania, 95124 Catania, Italy; andrea.zammitti@phd.unict.it (A.Z.); gsantisi@unict.it (G.S.); 2 Faculty of Human and Social Sciences, Kore University, 94100 Enna, Italy; angela.russo13@unikorestudent.it

**Keywords:** personal values, risk intelligence, career choices, job insecurity, uncertainty

## Abstract

In a risk society, personal values can be important resources, useful for managing uncertainty and guiding people in the perception of risk. The goal of this article is to explore the relationship between risk intelligence and personal values. The participants were 731 Italian adults aged between 18 and 65 years (M = 30.25; DS = 10.71). The survey was composed of the following measures: Subjective Risk Intelligence Scale and Portrait Values Questionnaire. Data analyses have found significant relationships between some types of personal values and risk intelligence: subjective risk intelligence is negatively related to conservation and positively related to openness to change and self-transcendence, but it was not related to self-enhancement. Furthermore, values of openness to change and self-transcendence mediate the relationship between age and subjective risk intelligence, while conservation values and self-enhancement values did not mediate the same relationship. Implication for practice and future research will be discussed.

## 1. Introduction

The nature of work has profoundly changed since the 20th century. In the past, the stability of organizations provided a solid base to build and design a long-term career path. Since the end of the second millennium, this security has been replaced by a new scenario that is characterized by flexible jobs that may generate anxiety and discomfort [1]. Economic changes have forced many companies to change their strategy [2], through automation, digital platforms, and other innovations. On the one hand, the evolution of technology has brought the promise of higher productivity (and with productivity, economic growth) and greater efficiency; on the other hand, it has raised difficult questions about the wider impact of automation on jobs, skills, wages, and the nature of work itself [3].

The lack of available jobs has also added to the changing nature of the work, even for those who keep their jobs: organizations tend to opt for fixed-term contracts rather than long-term employments [4,5,6]. Moreover, according to the March 2021 edition of the ESDE quarterly review, in most countries and across a broad range of sectors, self-employed workers have experienced particularly steep cuts in working hours and income [6].

All these changes affect a growing number of workers, causing a sense of insecurity, which has an objective and a subjective meaning. From an objective point of view, the concept of job insecurity refers to the threat of job loss emerging from the temporary nature of the job or the organization facing a merger, a downsize, or a reorganization [7], within a not-very-dynamic labour market. From a subjective point of view, the concept of job insecurity refers to a general concern about the continuity and stability of employment as it is currently experienced [8]. In this sense, it is possible to affirm that job insecurity can be considered a subjective phenomenon independent from the objective situation [7,9] because, even in the same seemingly objective environment, individual employees experience different levels of job insecurity [10,11].

It can be argued that workers react to the gradually changing characteristics of employment conditions and jobs [12], but this reaction is not automatic, and it depends on individual factors, such as age, gender, educational attainment, and locus of control; organizational factors, such as role ambiguity, role conflict, amount of organizational communication, and level of organizational change; and social, political, and cultural factors, such as country of origin [13]. Regarding the differentiation in workforce age, it can be argued that Millennials and Generation Y, more than Generation X and Baby Boomers, perceive that employers can be unreliable and, therefore, it is best that they do not over-commit to the organizations they work for, instead striving for personal development, with the goal of rapid career advancement rather than life-long employment [14]. Moreover, the perception of uncertainty does not seem to depend on the type of occupation; in fact, both less-skilled occupations (e.g., drivers, security personnel, and cleaners) and their highly skilled counterparts (e.g., lawyers, physicians, analysts, and managers) are increasingly vulnerable to job loss [15].

The framework of the Life Design paradigm [16] provides adequate constructs and methodologies for understanding how emerging adults and workers can face multiple career transitions and for supporting them in dealing with these challenges. Among psychological constructs, subjective risk intelligence could serve as a psychological resource that aims young adults and workers toward developing effective strategies in meaning-making with occurring events, so that uncertainty and risks could be managed in an effective rather than maladaptive way [17]. Given today’s globalized workforce, the Life Design paradigm offers useful resources to enable individuals to actively adapt and flourish in the working world [18].

In a world made up of multiple choices [19], people are confronted with the experience of uncertainty [20]. It becomes important to exercise the ability to define what may happen in the future and to choose among alternatives, the capacity to manage risk, and the appetite to take a risk and make forward-looking choices [21]. Then, according to Bernstein [21], it is necessary to reconsider the concept of risk, and recharacterize it as an opportunity; furthermore, it is necessary to consider the subjective dimension related to the perception of risk and not neglect the emotional components related to risk and their role in risk perception [22,23,24,25].

## 2. Literature Review: Subjective Risk Intelligence and Values

### 2.1. Subjective Risk Intelligence

The construct of subjective risk intelligence (SRI) has recently been introduced in career counselling [26], and it is defined as a multidimensional psychological characteristic that helps individuals to effectively evaluate risks, considering their advantages and disadvantages, approaching them as opportunities more than as threats, and feeling able to manage a certain margin of uncertainty about the outcomes of their choice [27]. Risk-intelligent individuals can effectively evaluate risky situations, considering them as opportunities and not threats; they develop a positive attitude in uncertain situations and face unpredictability with creativity and emotional control [27]. Nicholson and West [28] consider career changes as risky situations.

SRI is composed of the following dimensions: Imaginative capability refers to the individual’s ability to explore the unknown and produce new ideas [29,30] useful for reaching a goal [31,32,33,34]. Problem-solving self-efficacy is a dimension that includes self-confidence, confidence in one’s ability to manage situations, and the ability to make decisions; it positively influences the commitment that the individual puts into achieving his goals [35] and increases the feeling of being able to control risk [36]. Emotional stress management is related to the ability to modulate emotional responses in stressful situations; emotional stress can have negative effects on concentration, attention, work, and other important areas of functioning, while self-regulation is linked to various forms of risk-taking [37]. Lastly, attitude towards uncertainty refers to the ability to perceive uncertainty as an opportunity rather than a threat. The validation study of the scale and of the construct provides statistical evidence—through EFA, CFA, internal consistency, measurement invariance across gender, and concurrent validity—of its unique dimensional structure.

Risk-taking behaviour is positively associated with male gender [38], temporal orientation [39], and positive affective traits [40]. Additionally, self-efficacy shows a positive relationship with risk-taking [41], because it is a resource that allows people to better face uncertain situations [42]. Risk-taking behaviour is negatively related to openness to innovation [43] and positively related with creativity [44,45,46,47,48]. Finally, risk-taking behaviour is associated with self-regulation of emotions [37]. Risk intelligence showed a positive correlation with age and personal values [49].

Gender differences have emerged in some of these dimensions. For example, research has shown that men, compared to women, seem to better manage work-related stress [50], tend to experience negative emotions with less frequency [51,52,53], show fewer depressive and anxious symptoms [54,55,56], and have higher levels of self-efficacy in career choices related to disciplines such as mathematics and computer science [57,58,59,60,61]. Although there are not many studies on risk intelligence, based on previous research on similar dimensions, we can hypothesize gender differences in levels of risk intelligence.

### 2.2. Personal Values

Schwartz defines values as desirable states, objects, goals, or behaviours transcending specific situations and applied as normative standards to judge and to choose among alternative modes of behaviour [62]. Further specifying the construct, Harris [63] distinguishes values from goals, as the latter would describe something achievable, while the values would be intangible directions towards which to tend. Using a geographic metaphor, values are the cardinal directions, while goals are the specific coordinates of the place to be reached.

Schwartz [64,65] distinguishes 10 types of values: self-direction, stimulation, hedonism, realization, power, security, conformity, tradition, benevolence, and universalism. These values are in a dynamic relationship and their arrangement along two bipolar dimensions makes it possible to represent the opposition relations between conflicting values [64,65]. Specifically, the first dimension opposes the values of openness to change, which emphasize independent thought and actions (self-direction) and foster change (stimulation), to the values of conservation, which emphasize the protection of stability (security), the preservation of traditional practices (tradition), and the adaptation of one’s behaviour to conventional norms (conformity). The second dimension opposes the values of self-transcendence, which emphasize acceptance of others as equals (universalism) and concern for their well-being (benevolence), to the values of self-enhancement, which emphasize the pursuit of one’s relative success (achievement) and dominance over others (power). Moreover, hedonism has aspects linked both to openness to change and to self-improvement [66].

Values are related to choice behaviours [67]. In the literature, some authors hold that values guide behaviour and even include this guiding role in their definition of values [68,69]. Others conclude that values rarely guide behaviour and do not for most people [70,71]. To be precise, values can direct and influence career choices [72]. Super [73] considers values as the basis for the activities and roles that people assume in their professional career. Indeed, individuals rely on their values, at least in part, when choosing the occupation or profession for which they wish to prepare [74]; for example, Feather [75] has shown that they predicted a choice between university courses. According to Feather [76,77,78,79], the values act in the selection processes as they determine the attractiveness of the results: by this logic, if a student had to choose between going out or studying for a competition, he would choose based on the most important value, either ambition or friendship in this case [80]. Moreover, some careers can be typified by the values people consider more important for them or by the values they depreciate, or by both [72]. Noteworthy is the finding that congruency between people’s values and their work environment is related to work satisfaction [81].

## 3. Rationale of the Study

The main assumption behind this study is that the action of taking risks is not something that endangers the safety of persons; rather, the assumption of risk is considered an adaptive dimension of the action. Values can activate resources to deal with uncertainty [82]. Risk intelligence and its dimensions can be related to values.

Imaginative capability, one of the dimensions of risk intelligence, is linked to creativity, as both refer to the possibility of creating something new [83,84]. According to Gaut [85], imagination can be considered a vehicle for active creativity. Indeed, while imagination involves thinking about something that does not exist, creativity refers to creation, putting into practice what has been thought [86]. Some researchers have found that creativity correlates negatively with conservation values and positively with the values of independence and autonomy [87,88].

Similarly, in situations of uncertainty, the probability of something happening is not measurable; in other words, outcomes are known but the probabilities of their occurrence are unknown [89]. These situations could require a positive attitude towards uncertainty and openness to change.

Furthermore, self-efficacy is a dimension linked to success, as people who have high levels of self-efficacy are committed to achieving their goals [90], and some research has shown that they are successful in various areas [91].

Finally, according to Bachkirova [92], personal values play an important role in the occurrence of stress: the value of obtaining or maintaining a high position influences self-esteem and can bring satisfaction or stress. Moreover, emotional self-regulation is linked to various forms of risk-taking [37].

There are not many studies that have investigated the effect of age diversity on risky choices. Some authors [93] have found that younger managers have a greater propensity to make risky decisions, probably to show off their skills [94]; older ones, on the other hand, prefer lower risk due to economic threats [95,96]. However, when career concerns are prevalent, younger managers are more risk-averse as they experience greater uncertainty about their future careers than older ones [97]; the latter have no career worries thanks to their accumulated human capital [98].

Similarly, the similarity-attraction paradigm [99] suggests that individuals born at similar times will most likely develop similar views on their life experiences. This means that similar generations will have similar values [100] and age difference leads to variations in personal values [101,102,103]; this, in turn, causes the generational difference between young and old [104]. In the study by Talavera, Yin, and Zhang [105], it was found that six value indicators (risk, prudence, wealth, success, creativity, and slowness) are significantly influenced by age. Additionally, age diversity can negatively impact an organization’s profitability through values.

## 4. Aims of the Study

Starting from the rationale, this study aims to explore the relationship between values and risk intelligence. The theoretical premises lead us to think that certain values could help people in managing uncertainty. Therefore, we hypothesize that:

**Hypothesis** **1a** **(H1a).** *Values of openness to change positively affect risk intelligence*;

**Hypothesis** **1b** **(H1b).** *Self-enhancement values negatively affect risk intelligence*;

**Hypothesis** **1c** **(H1c).** *Self-transcendence values positively affect risk intelligence*;

**Hypothesis** **1d** **(H1d).** *Conservation values negatively affect risk intelligence*;

**Hypothesis** **2a** **(H2a).** *Openness-to-change values mediate the relationships between age and risk intelligence*;

**Hypothesis** **2b** **(H2b).** *Self-enhancement values mediate the relationships between age and risk intelligence*;

**Hypothesis** **2c** **(H2c).** *Self-transcendence values mediate the relationships between age and risk intelligence*;

**Hypothesis** **2d** **(H2d).** *Conservation values mediate the relationships between age and risk intelligence*.

## 5. Materials and Methods

### 5.1. Participants

The data were collected through an online survey; moreover, we used convenience sampling from the general population. The participants were 731 Italians, aged between 18 and 65 years (Mean, M = 30.25; Standard Deviation, SD = 10.71), all volunteers. Participants completed the test individually and anonymously, after having expressed their consent to participation, with the following instructions: “In the following survey you will find some statements describing behaviours, situations or ways of thinking. Indicate the choice that best expresses how much you feel in agreement with each of them. Select one answer for each statement. There are no right or wrong answers. The data will be processed in aggregate form and in no way will it be possible to trace the characteristics of the individual participant; therefore, the anonymity of the persons involved is guaranteed, in absolute respect of the laws on the protection of privacy. We are interested in knowing only the gender, age, professional role or some data related to the job position. Please answer all questions as sincerely as possible. Thanks for the collaboration. Good job!”. The participants were free to abandon the test at any moment.

### 5.2. Measures

#### 5.2.1. Personal Information

Participants provided personal information including age, gender and educational attainment.

#### 5.2.2. Risk Intelligence

The Subjective Risk Intelligence Scale (SRIS, [27]) has a total of twenty-one items; the statements presented describe behaviours or moods, and respondents have to answer in a five-point Likert-type scale from 1 (totally disagree) to 5 (totally agree). Sample items are “to be able to create new procedures, I think for myself instead of following procedures established by others” (measure of imaginative capability), “I feel able to make decisions even when I don’t have all the information” (measure of problem-solving self-efficacy), “when I feel fearful about something, I have difficulty concentrating on everything” (measure of emotional stress vulnerability), and “the uncertainty about possible developments of a situation paralyzes me” (measure of negative attitude toward uncertainty). The scale allows calculating a total subjective risk intelligence score. In this study, Cronbach’s alpha was 0.89.

#### 5.2.3. Values

The Portrait Values Questionnaire (PVQ, [106,107]) has a total of forty items based on the descriptions of different people with two sentences. For example, “It is important for him/her to be rich. He is afraid to have a lot of money and expensive things” (measure of power value). The subject must answer the question “how much like you is this person’’ by using a six-point scale. The possible answers are very similar, similar, rather similar, rather dissimilar, dissimilar, and very dissimilar. The forty items measure ten values; they are grouped into four distinct areas by Schwartz [64]. In this study, all the Cronbach’s alpha values are between 0.77 and 0.81.

### 5.3. Data Analysis

Statistical analysis of the data was conducted using the RStudio software version 1.2.5033 [108,109]. RStudio is an Integrated Development Environment (IDE) for the R programming language [110]. It allows you to carry out a variety of statistical analyses through the use of additional packages that are available on the Comprehensive R Archive Network (CRAN) website. The level of significance for all analyses was set at α = 0.05. The Shapiro–Wilk test was used to verify if variables were normally distributed. Most of the variables were not normally distributed. Spearman’s non-parametric correlations were conducted. The Mann–Whitney U test was used to test if the data collected from males and females differed significantly.

The mediation analysis was verified using the Mediation package. To examine whether values mediate the relationship between age and risk intelligence, we performed regression-based mediation analysis by estimating all the pathways and using the procedures indicated by Preacher and Hayes [111]. Through this approach, it is possible to verify the mediation by evaluating the statistical significance of the indirect effect, that is, the path that goes from the independent variable to the dependent variable through the mediator variable. To obtain the confidence intervals for the indirect effect, a bootstrap procedure was used; this procedure involves repeated sampling from the original sample to create an empirical approximation of the sample distribution of the indirect effect. According to the approach of Preacher and Hayes [96], if the bootstrap confidence interval does not include zero, the mediating effect can be considered significant. This procedure has the advantage of being independent of the distributional assumptions relating to the parameter estimates for the indirect path. Our analyses were based on 5000 bootstrap samples, following the indications of Preacher and Hayes [112].

## 6. Results

### 6.1. Preliminary Analysis

Descriptive characteristics of the sample are found in Table 1. The normality of the data distribution for the scales was tested using the Shapiro–Wilk test. The Shapiro–Wilk test for normality showed that there were significant deviations from normality in the investigated dimensions for all constructs (*p* < 0.05). No data transformations were conducted.

Bivariate correlations between the variables included in our models are presented in Table 2.

### 6.2. Mediation Model

**Figure 1:**
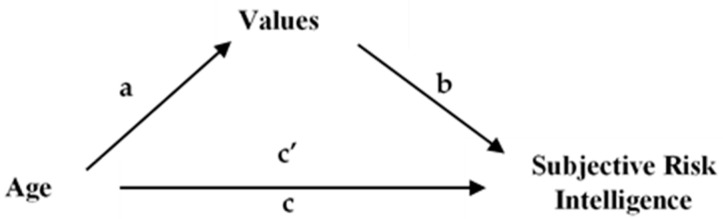
Mediation model. Note: a = effect of age on values (openness to change, self-transcendence, self-enhancement, conservation); b = effect of values on risk intelligence; c = total effect of age on risk intelligence when values are not included as mediators; c’ = direct effect of age on risk intelligence when values are included as mediator.

To examine the influence of values on risk intelligence, four multi-mediated analyses were conducted ([113,114], model 6). Our proposed mediation model consists of the following components (see Figure 1): age constituted the independent variable, values were introduced as the mediator variable, and subjective risk intelligence was the dependent variable.

The results of the mediations are presented in Table 3, which contains the standardized *β*, indicating the intensity of the effect, and the 95% confidence intervals (CI), indicating the significance of the effect with a 5% probability of error (CI that do not contain 0 are significant). The results show that, consistent with H2, the hypothesized total model is significant (*β* = 0.01, CI = 0.00 to 0.01). Age had a direct effect on subjective risk intelligence (*β* = 0.02, CI = 0.01 to 0.02).

Regarding path a, age had a direct effect on openness to change values (*β* = −0.13, CI = −0.14 to −0.00), self-transcendence values (*β* = 0.13, CI = 0.00 to 0.01), and self-enhancement values (*β* = −0.08, CI = −0.13 to −0.00); in contrast, age had no effect on conservation values.

The following effects were significant regarding path b: openness to change (*β* = 0.37, CI = 0.22 to 0.33), self-transcendence (*β* = 0.17, CI = 0.08 to 0.20), and conservation (*β* = −0.21, CI = −0.22 to −0.10); conversely, self-enhancement values did not have effects on subjective risk intelligence.

Moreover, results show the indirect effect of age on subjective risk intelligence mediated by openness to change (*β* = −0.00, CI = −0.01 to −0.00) and self-transcendence (*β* = 0.00, CI = 0.00 to 0.00).

No mediating effect was found between age and subjective risk intelligence for conservation values and self-enhancement values.

## 7. Discussion

In this study, we aimed to explore how values could influence risk intelligence, conceptualized as a multidimensional psychological characteristic that helps individuals to effectively assess risks, considering their advantages and disadvantages, facing them as an opportunity rather than a threat, and feeling able to manage a certain margin of uncertainty about the outcome of one’s choice [27].

We firstly hypothesized that values of openness to change (H1a), self-enhancement (H1b), and self-transcendence (H1c) were positive antecedents of subjective risk intelligence; following the same reasoning, we hypothesized that values of conservation (H1d) were negative antecedents of risk intelligence. These assumptions have been partially confirmed, as it was seen that subjective risk intelligence was negatively related to conservation (H1d) and positively related to openness to change (H1a) and self-transcendence (H1c) but was not related to self-enhancement (H1b).

Regarding our hypothesis 1a, it is known that risk orientation is a very important aspect of managerial culture, and it has been studied while treating openness as a personality trait [115,116]. We wanted to extend these studies by considering openness as a value, as this attitude towards innovation has been related to risk-taking by other studies [117]. Our results show that the values linked to novelties, challenges, and independent thinking are also linked to the ability to see risk as an opportunity. Risk-taking has been studied as a characteristic of managers [117], and it has been found that managers show the ability to manage and exercise their power and that self-improvement values are positively linked to leadership [118]. This led us to speculate that self-improvement values could also be related to risk intelligence (H1b). Our hypothesis has not received confirmation. This is probably due to the random sampling of the research, which does not consider the type of work that the participants perform.

As regards our H1c, it appears that those with higher levels of self-transcendence values, emphasizing acceptance of others as equals and concerning for their well-being, may have higher levels of subjective risk intelligence. This result could suggest that social support, which is often shown by those with high levels of universalism and benevolence values [119], could mediate between self-transcendence and risk intelligence; further studies would be needed to explore this relationship.

Regarding our hypothesis 1d, it has been confirmed that subjective risk intelligence is negatively related to high levels of conservation values; people who emphasize the protection of stability (security), the preservation of traditional practices (tradition), and the adaptation of one’s behaviour to conventional norms (conformity, appear to have lower levels of subjective risk intelligence. Considering that people with high traditional values suffer more health consequences from job insecurity [120], they may tend to avoid risks, developing less risk intelligence. In fact, it is known that the “adapted”, compared to the “innovators”, have lower scores in risk-taking, preferring risk avoidance [121].

The relationship between age and risk has been explored several times [122,123]. Even using both self-report and behavioural measures to evaluate risk-taking, it has been found that there is a decline in the propensity for risk across the lifespan [124]. A development explanation would argue that being younger contributes to predict higher levels of risk-taking because the developmental track, along which people move as they increase in age, leads them to take fewer risks: as a person gets older, gets married, has children, and acquires responsibilities to persons other than himself/herself that oppose his taking risks [121]. If the relationship between age and risk-taking is influenced by specific characteristics of the task [122], what influences the relationship between age and subjective risk intelligence?

Our second group of hypotheses claimed that values of openness to change (H2a), self-enhancement (H2b), self-transcendence (H2c) and conservation (H2d) would mediate the relationships between age and subjective risk intelligence. The results show that values of openness to change and self-transcendence mediate the relationship between age and subjective risk intelligence, while conservation values and self-enhancement values did not mediate the same relationship. These might suggest that the emphasis on one’s independent thinking and actions such as readiness for change (H2a; [123]) and the tendency to preserve and improve the well-being of all people and nature (H2c; [123]) can predispose to a greater ability of people, to feel able to manage a certain margin of uncertainty about the outcome of their choice, both emotional and design, following an effective evaluation of the advantages and disadvantages of possible choices. Therefore, preparing for change is useful not only to develop greater self-efficacy in the workplace [124], but also to develop greater self-efficacy towards choices perceived as risky, regardless of age. Furthermore, consistent with Reed’s [125] theory, which conceives self-transcendence as embodying “experiences that connect rather than separate a person from self, others, and the environment”, as a “development imperative” and as a basic resource for “realizing one’s potential for well-being” ([125] p. 111), the values of self-transcendence could predispose a better ability to perceive risk as an opportunity within which to make creative decisions, modulating emotional responses in contexts of uncertainty [27].

Following the results that disconfirm our third hypotheses, from the one side, self-enhancement values (H2b), consistently with the contradiction of our hypothesis 1d and not having a direct effect on risk intelligence (path a), do not mediate the relationship between age and risk intelligence; on the contrary, conservation values (H2d), despite having a direct effect on risk intelligence (path b), are not influenced by age (path a), as opposed to what has been found in other studies [126,127]. This could probably be due to specific characteristics of our convenience sample.

## 8. Conclusions and Limitations

The society of the third millennium requires the ability to define what could happen in the future and to choose between alternatives in an uncertain context [27]. In this variable framework, job insecurity reflects a threat to the continuity and stability of employment as it is currently experienced [8] and it depends on several factors as microeconomic and social environments [128], organizational practices [129,130,131], and individual characteristics, conditions, and resources as negative affectivity [132], self-esteem [133], external employability [134,135], career adaptability [136], personal values [120] and so on.

Within this background, the feeling of risk arises from the perception of different aspects of decision making and is driven by either the fear of losing (affordable loss) or the desire to win (return maximization) [137,138,139]. In this sense, Bernstein [21] argues that the ability to manage risk and take risks to make forward-looking choices is a key skill. Such choices can be influenced by what is important to people, that is, by personal values [69].

The results of the present study can be read in the frameworks of positive psychology. Openness to change and self-transcendence values can help people manage uncertainty in the context of ongoing technological, social, economic and political changes in the world of work. Our study shows how being guided by values of openness to change and self-transcendence can predispose to a greater ability to evaluate the advantages and disadvantages of the present choice alternatives, managing emotions and promoting effective and creative problem solving; on the contrary, people guided by conservation values, which emphasize the protection of stability, the preservation of traditional practices and the adaptation of one’s behaviour to conventional norms tend to have lower levels of risk intelligence. Moreover, being guided by values of openness to change and self-transcendence mediates the relationship between age and risk intelligence.

Regarding the limitations of the present study, these could include: firstly, the cross-sectional design of the study, which does not allow causal relationships to be established and inverse causality to be excluded; secondly, the use of self-report tools, which can be affected by social desirability; and thirdly, the choice of a convenience sampling that does not allow to consider the study’s sample as representative of the general population. Moreover, a concern arising in studies with single-source, self-report, and cross-sectional designs is common method bias, since the same method is used to measure multiple constructs. Finally, the lack of a behavioural dimension and data on the outcomes of risky choices represents a limit in the explanation of the model.

## 9. Implications for Future Studies and Practice

Future longitudinal studies could explore the relationships between values and different dimensions of risk intelligence. A useful direction for future research could be to examine how the values of openness to change, self-transcendence, self-enhancement and conservation influence the dimensions of imaginative capability, emotional stress vulnerability, problem-solving self-efficacy, and attitude towards uncertainty in different groups of workers. Along these lines, research might yield useful insights into why people with a growth orientation are more risk-avoidant [140], although our study does not show that higher levels of self-enhancement predict lower levels of risk intelligence. Moreover, considering that self-improvement values are positively linked to leadership [118] and that self-affirmations may sometimes reduce the motivation of people to reach toward their most ambitious aspirations, decreasing perceived self-efficacy [141], it could be interesting to explore whether problem-solving self-efficacy could be the dimension that causes managers guided by self-improvement values to have lower levels of risk intelligence.

Finally, in the context of the changing nature of the 21st-century world of work, considering that mental and physical health costs of job insecurity could be high [142] and that daily value-based actions are related to greater daily psychological well-being and lower daily psychological distress [143], it is important to include the exploration of one’s personal values within educational and professional guidance programs that aim to promote risk intelligence as a useful resource for career adaptability and career development. Indeed, career counsellors should be concerned with the promotion of personal resources in different contexts and educational levels [144,145,146]. Clarity about personal values is a core dimension of psychological flexibility [63] and, therefore, can support effective actions in the direction of what people want to achieve, helping them to move in contexts of uncertainty.

## Figures and Tables

**Table 1:** Characteristics of the sample.

Dimensions	Descriptives
Age, M (SD)	30.25 (10.71)
Gender, *n* (%)	
male	342 (46.8)
female	389 (53.2)
Educational attainment, *n* (%)	
middle school	45 (6.2)
high school graduates	351 (48.0)
graduates	335 (45.8)
Risk intelligence, M (SD)	3.42 (0.56)
Openness to change, M (SD)	4.34 (0.74)
Self-transcendence, M (SD)	4.83 (0.71)
Self-enhancement, M (SD)	3.63 (0.92)
Conservation, M (SD)	4.08 (0.70)

**Table 2:** Bivariate correlations between model variables (Spearman’s rho).

Variables	1	2	3	4	5	6
1. Age	‒					
2. Risk intelligence	0.282 **	‒				
3. Openness to change	−0.101 **	0.360 **	‒			
4. Self-transcendence	0.079 *	0.236 **	0.295 **	‒		
5. Self-enhancement	−0.089 *	0.127 **	0.403 **	0.043	‒	
6. Conservation	0.029	−0.058	0.157 **	0.395 **	0.252 **	‒

Note: * *p* < 0.05, ** *p* < 0.01.

**Table 3:** Mediation of the effects of age on risk intelligence through values (standardized *β*).

Dimensions	Mediation Analysis				Indirect Effect		
	Path a		Path b		Point estimate	CI	
	*β*	CI	*β*	CI	*β*	Lower	Upper
Openness to change	−0.13	−0.14 to −0.00	0.37	0.22 to 0.33	−0.00	−0.01	−0.00
Self-transcendence	0.13	0.00 to 0.01	0.17	0.08 to 0.20	0.00	0.00	0.00
Self-enhancement	−0.08	−0.13 to −0.00	0.05	−0.01 to 0.07	−0.00	−0.00	0.00
Conservation	0.01	−0.00 to 0.01	−0.21	−0.22 to −0.10	−0.00	−0.00	0.00

## Data Availability

The data will be provided upon request to the corresponding author.
